# Efficacy of Transcranial Alternating Current Stimulation for Major Depressive Disorder and the Role of Intensity: A Systematic Review and Exploratory Meta-Analysis

**DOI:** 10.31083/AP47383

**Published:** 2026-07-31

**Authors:** Li Qi, Yue Yu, Xiaomin Pan, Jin Li, Zhishun Gao, Ting Zhang, Wenjia Wang, Kai Wang, Qianqian Li, Tongjian Bai

**Affiliations:** ^1^Department of Neurology, The First Affiliated Hospital of Anhui Medical University, 230022 Hefei, Anhui, China; ^2^Department of Psychology and Sleep Medicine, The Second Affiliated Hospital of Anhui Medical University, 230601 Hefei, Anhui, China; ^3^Department of Psychiatry, The First Affiliated Hospital of Anhui Medical University, 230022 Hefei, Anhui, China; ^4^Department of Neurology, The Second Affiliated Hospital of Anhui Medical University, 230601 Hefei, Anhui, China; ^5^Institute of Artificial Intelligence, Hefei Comprehensive National Science Center, 230088 Hefei, Anhui, China; ^6^Anhui Province Key Laboratory of Cognition and Neuropsychiatric Disorders, 230032 Hefei, Anhui, China; ^7^Collaborative Innovation Center of Neuropsychiatric Disorders and Mental Health, 230032 Hefei, Anhui, China; ^8^Anhui Institute of Translational Medicine, 230032 Hefei, Anhui, China; ^9^The School of Mental Health and Psychological Sciences, Anhui Medical University, 230032 Hefei, Anhui, China

**Keywords:** major depressive disorder, electric stimulation therapy, transcranial alternating current stimulation, safety

## Abstract

**Background:**

Current treatments for major depressive disorder (MDD) are limited by efficacy and adverse effects. Transcranial alternating current stimulation (tACS) has emerged as a promising alternative. However, there is still a lack of systematic evaluation of its effectiveness. Recent studies suggest that currents greater than 7 milliamperes (mA) may be necessary to significantly alter potentials in deep brain regions associated with depression. We conducted a systematic review and exploratory meta-analysis to assess the overall efficacy and safety of tACS for MDD and to explore potential associations regarding current intensity by comparing high-intensity tACS (HI-tACS) and low-intensity tACS (LI-tACS) protocols.

**Methods:**

A systematic search of Embase, PubMed, Web of Science, the Cochrane Library, ClinicalTrials.gov, and WHO ICTRP was conducted from inception to January 5, 2026. Data were analyzed using a random-effects model, assessing changes in depressive scale scores, response and remission rates, discontinuation, and adverse events.

**Results:**

Randomized controlled trials with 438 participants were included. Compared to sham stimulation, active tACS was associated with a significant reduction in depressive symptoms (moderate effect size). In an exploratory subgroup analysis, HI-tACS was associated with a larger effect size (large effect) than LI-tACS (small effect), with lower heterogeneity observed within subgroups. The HI-tACS group also showed significantly higher response and remission rates. No significant differences in adverse events or treatment discontinuations were observed between the two subgroups.

**Conclusions:**

Our results suggest that tACS may be a potentially effective treatment for MDD. Data from exploratory subgroup analyses provide preliminary, hypothesis-generating evidence that higher intensity may be associated with improved outcomes. However, given the limited evidence base and substantial heterogeneity, as well as the fact that intensity co-varied with stimulation frequency and other parameters in the included trials, these findings do not establish a sole causal link for intensity. Future large-scale, controlled, multi-arm trials are needed to disentangle these factors and confirm these preliminary observations.

**The PROSPERO Registration:**

The study has been registered on https://www.crd.york.ac.uk/prospero/ (registration number: CRD42024589889; registration link: https://www.crd.york.ac.uk/PROSPERO/view/CRD42024589889).

## Main Points

1. High-intensity transcranial alternating current stimulation (HI-tACS, >7 milliamperes [mA]) was associated with a large therapeutic effect for major depressive disorder (MDD), whereas low-intensity protocols showed a small effect.

2. The greater effect of high-intensity stimulation aligns with biophysical evidence suggesting that currents >7 mA may be required to modulate deep brain structures.

3. High-intensity tACS was generally well-tolerated, with no significant subgroup differences in adverse events or discontinuation rates between the two intensity subgroups.

4. Current intensity emerged as a potential moderator of treatment outcomes, suggesting a need for physiologically grounded dosing in future trials.

## 1. Introduction

Major depressive disorder (MDD) is a prevalent and severe mood disorder [1], characterized by persistently low mood, diminished interest or pleasure in activities. Despite the critical need for effective interventions, MDD treatment remains challenging [2]. Clinical guidelines primarily recommend pharmacotherapy and psychotherapy as first-line treatments [3]. However, a significant proportion of patients derive limited benefits from pharmacotherapy or psychotherapy. A meta-analysis of 165 placebo-controlled trials indicates that only 54% of adults demonstrate improvement with antidepressant medication, with placebo response rates ranging from 35% to 40% [2,4]. Moreover, pharmacotherapy is frequently associated with adverse effects, such as weight gain, sexual dysfunction, and gastrointestinal disturbances [5]. Psychotherapeutic approaches, while effective, are time-intensive and costly, with access further hindered by cultural and linguistic barriers, societal stigma, and a shortage of trained therapists [6].

As an alternative, non-invasive brain stimulation (NIBS) has demonstrated efficacy in treating depressive symptoms and a favorable side effect profile, thereby attracting increasing attention in recent years [7]. Transcranial alternating current stimulation (tACS) is a rapidly evolving NIBS modality that not only modulates immediate cortical excitability but also induces lasting neuroplastic changes in the human cortex [8,9,10]. By delivering weak alternating currents to the scalp, tACS entrains endogenous neural oscillations, theoretically offering a mechanism-based intervention for the dysrhythmias observed in psychiatric conditions [11,12,13]. tACS offers a more accessible alternative to other NIBS techniques, such as transcranial magnetic stimulation (TMS), due to its portability and cost-effectiveness. However, the therapeutic application of tACS has been hampered by significant heterogeneity in efficacy across studies [14], which limits the interpretability of existing evidence and hinders its broader clinical application [15]. While such heterogeneity in psychiatric clinical trials is often attributed to geographical and cultural factors affecting placebo response or symptom reporting [16], we propose that fundamental biophysical parameters may play a more decisive role.

Specifically, converging lines of evidence from biophysical modeling and animal physiology suggest that the electric fields generated by conventional low-intensity protocols (<2 milliamperes [mA]) might be insufficient to reliably entrain neuronal spiking in human circuits [17]. Intracranial measurements suggest that an electric field strength of approximately 1 V/m is likely required to effectively bias spike timing [18]. However, due to significant current shunting by the scalp and skull, conventional intensities are estimated to induce peak fields of only 0.2–0.8 V/m in the human brain [19]. This attenuation is thought to be particularly relevant for deep brain structures, such as the amygdala and hippocampus, which are widely considered to play a critical role in the pathophysiology of MDD [20,21,22,23,24]. Finite element modeling (FEM) and intracranial measurements suggest that these deep targets receive substantially weaker electric fields compared to superficial cortical regions [25]. Furthermore, *in vivo* studies in non-human primates have demonstrated a linear dose-response relationship, implying that higher intensities may be necessary to maximize neuronal entrainment without reaching a saturation plateau [26,27]. Consistent with these preclinical models, a recent study utilizing stereoelectroencephalography (SEEG) in humans provided preliminary evidence that alternating currents of at least 7 mA were needed to elicit significant voltage changes in these deep limbic targets [28]. Collectively, these findings support the hypothesis that stimulation intensity could be a key variable influencing clinical efficacy.

Based on these physiological lines of evidence, we hypothesize that insufficient current intensity may represent a key contributor to trial failure. We conducted this exploratory meta-analysis to examine whether stratifying studies by the 7 mA physiological threshold could reduce statistical heterogeneity. Furthermore, to ensure the reliability of our findings, given the limited number of trials, we strictly adhered to methodological standards by avoiding the double-counting of patient populations from the same research centers, a common source of bias that artificially inflates precision in meta-analyses [29,30].

## 2. Material and Methods

### 2.1 Study Overview

The meta-analysis was conducted in accordance with the Preferred Reporting Items for Systematic Reviews and Meta-Analyses (PRISMA) guidelines (**Supplementary Table 1**). The protocol was registered with the International Prospective Register of Systematic Reviews (PROSPERO, https://www.crd.york.ac.uk/PROSPERO/, registration number CRD42024589889). Data reporting adhered to the Cochrane Handbook for Systematic Reviews of Interventions [31]. Formal ethical approval was not required, as no primary data were collected.

### 2.2 Eligible Criteria

Studies were selected based on the Participants, Intervention, Comparison, Outcomes, and Study Design (PICOS) framework. To ensure the validity, rigor, and reproducibility of the study selection process, specific operational criteria were defined as follows. Inclusion Criteria: Participants: adults (age ≥18 years) with a primary diagnosis of MDD established by standardized diagnostic criteria, including the Diagnostic and Statistical Manual of Mental Disorders (DSM-IV, DSM-IV-TR, or DSM-5) or the Mini International Neuropsychiatric Interview (MINI). Intervention: active tACS applied to the head, irrespective of stimulation intensity, frequency, montage, or session duration, with or without adjunctive pharmacotherapy. Comparison: a sham tACS control group (sham stimulation). Outcomes: studies reporting sufficient data to calculate the primary outcome (change in depressive scale scores from baseline to endpoint). Secondary outcomes of interest included response rates (defined as ≥50% reduction in baseline scores), remission rates (e.g., Hamilton Depression Rating Scale (HDRS) score <7), discontinuation rates, and adverse events. Study Design: randomized controlled trials (RCTs). Exclusion Criteria: Population: studies involving patients with bipolar disorder, psychotic depression, or depression secondary to other medical or neurological conditions, or studies focused on adolescent populations (age <18 years). Intervention: studies involving single-session stimulation focused solely on immediate physiological mechanisms rather than clinical therapeutic efficacy. Study Design: non-randomized studies, observational studies, case reports, reviews, protocols, and conference abstracts without full data. Data Integrity: to ensure the independence of observations, overlapping datasets or secondary analyses derived from the same patient population were rigorously excluded to prevent double-counting [29,30].

### 2.3 Search Strategy

A systematic literature search was conducted across three major bibliographic databases: Embase (https://www.embase.com), PubMed (https://pubmed.ncbi.nlm.nih.gov), and Web of Science (https://www.webofscience.com), and three clinical trial registers: the Cochrane Library (https://www.cochranelibrary.com), ClinicalTrials.gov (https://clinicaltrials.gov), and WHO ICTRP (https://trialsearch.who.int). The search was initially performed on October 8, 2024, and the final updated search was conducted on January 5, 2026. To ensure comprehensive retrieval, the strategy employed a combination of controlled vocabularies (e.g., MeSH for PubMed, Emtree for Embase) and free-text terms. In addition, manual searches were performed using Google Scholar (https://scholar.google.com) and reference lists of identified articles were reviewed to ensure comprehensive inclusion. The detailed search strings for each database are presented in **Supplementary Table 2**.

### 2.4 Data Extraction

All potentially relevant citations were imported into EndNote 20 (Clarivate Analytics, Philadelphia, PA, USA) for management and de-duplication [32]. Two investigators (L.Q. and Y.Y.) independently screened the titles and abstracts of the identified records according to predefined inclusion and exclusion criteria. Disagreements were resolved through discussion or consultation with a third investigator (X.P.). Subsequently, the full texts of potentially relevant articles were reviewed. For each included study, the following data were extracted: the name and country of the first author, year of publication, diagnostic criteria, number and characteristics of participants, details of the stimulation protocol and sessions, and information pertaining to the outcomes. Furthermore, we documented the number of participants who discontinued treatment and those who experienced any adverse effects [33]. If changes in depressive scale scores were not directly reported in the articles, the mean change was calculated by subtracting the baseline score from the endpoint or follow-up score. The standard deviation was then derived according to the formula outlined in the Cochrane Handbook [31]. To ensure a conservative estimate and avoid overstating the precision of the change values, a correlation coefficient (Corr) of 0.2 was imputed for studies that did not report one.


$${\text{SD}}_{\text{change}}=\sqrt{{\text{SD}}_{\text{baseline}}^{2}+{\text{SD}}_{\text{final}}^{2}-2\times \text{Corr}\times {\text{SD}}_{\text{baseline}}\times {\text{SD}}_{\text{final}}}$$

### 2.5 Data Synthesis

All statistical analyses were conducted using RevMan 5.4.1 (The Cochrane Collaboration, London, UK). For continuous outcomes, standardized mean differences (SMDs) and 95% confidence intervals (CIs) were calculated. For dichotomous outcomes, risk ratios (RRs) with corresponding 95% CIs were computed. Statistical heterogeneity among studies was evaluated using the Cochran’s Q test (with *p* < 0.10 indicating significance) and the I^2^ statistic. I^2^ values of <25%, 25–50%, and >50% were interpreted as indicating low, moderate, and high heterogeneity, respectively [34]. Given the inherent clinical heterogeneity in tACS protocols and patient characteristics, a random-effects model was employed for all analyses to provide a conservative estimate of the pooled effect. To investigate potential sources of heterogeneity, subgroup analyses were pre-planned based on current intensity. To address potential unit-of-analysis errors arising from multi-arm trials with a shared control group, we employed the method of splitting the shared control group in our sensitivity analyses. This involved dividing the sample size and event counts of the shared control group approximately equally among the active intervention arms, as recommended by the Cochrane Handbook [31]. Subsequently, as a secondary exploratory analysis, subgroups were defined according to the different current intensities applied to compare low-intensity (LI)-tACS and high-intensity (HI)-tACS. The threshold of >7 mA for HI-tACS was chosen based on recent evidence from a study using SEEG, which provided a physiological rationale by suggesting that currents of at least 7 mA are necessary to directly and significantly modulate local field potentials in deep brain regions implicated in depression [28]. We caution that this threshold serves as an exploratory operationalization for this meta-analysis rather than a universally applicable biological constant. The actual intracranial electric field intensity depends on the specific electrode montage and the volume conduction properties of the scalp and skull [35,36]. Statistical significance was set at a threshold of *p* < 0.05. SMD values were categorized as follows: <0.40 indicating a small effect, 0.40–0.80 indicating a moderate effect, and >0.80 indicating a large effect. Potential publication bias was assessed by visual inspection of funnel plots.

### 2.6 Assessment of Study Quality

The risk of bias in RCTs was assessed using the Cochrane Collaboration’s tool for assessing risk of bias [31,37]. This tool classifies studies based on their risk of bias, categorized as low, unclear, or high, across seven specific domains: random sequence generation, allocation concealment, blinding of participants and personnel, blinding of outcome assessment, incomplete outcome data, selective reporting, and other potential biases. Two independent reviewers evaluated the risk of bias for each study. Any discrepancies were resolved by discussion or consultation with a third investigator.

## 3. Results

### 3.1 Study Inclusion

**Supplementary Fig. 1** presents the PRISMA flow diagram outlining the process of literature screening and study selection. The initial search yielded a total of 750 records: 663 from bibliographic databases and 87 from registers. Manual searches in Google Scholar yielded 1 additional record. After removing duplicates and screening titles/abstracts, 21 full-text articles were assessed for eligibility. Specifically, none met the inclusion criteria in the search of registries as they were predominantly single-session mechanism studies, terminated trials, or duplicates of published datasets (see **Supplementary Fig. 1**). Consequently, four independent RCTs met the strict inclusion criteria. Although our comprehensive search initially identified six potentially relevant RCTs, a detailed inspection of participant overlap revealed that two earlier reports [38,39] originated from the same research center and involved patient populations overlapping with a more comprehensive subsequent trial. To prevent the inflation of precision and type I error associated with double-counting [29], these overlapping reports were excluded from the quantitative synthesis [30]. Consequently, four independent RCTs met the strict inclusion criteria [40,41,42,43].

### 3.2 Study Characteristics

A comprehensive summary of the study characteristics is summarized in Table 1 (Ref. [40,41,42,43]). Of the four articles, two are classified as LI-tACS and two as HI-tACS. One of the LI-tACS studies is a three-arm trial that compares 10 hertz (Hz) tACS, 40 Hz tACS, and a control group [40]. The other three studies are two-arm trials. The meta-analysis involved a total of 438 participants, with 221 in the intervention groups and 217 in the control groups.

**Table 1.** **Main characteristics of the included studies**.

Study		Country	Diagnostic Criteria	Age (years) Gender: Male/Female (n)		Stimulation protocol Site Frequency Intensity	Sessions	Concomitant Treatment
				Intervention	Control			
LI-tACS								
	Alexander et al. (2019) (a) [40]	USA	MINI	36.3 (15.2) 1/9	38.4 (13.5) 2/9	Anode: left and right frontal areas (F3 and F4) Cathode: Cz 10 Hz 2 mA at Cz and an amplitude of 1 mA at F3 and F4	40 min/day, 5 consecutive days	Stable Antidepressants Allowed
	Alexander et al. (2019) (b) [40]	USA	MINI	35.4 (11.6) 2/9	38.4 (13.5) 2/9	Anode: left and right frontal areas (F3 and F4) Cathode: Cz 40 Hz 2 mA at Cz and an amplitude of 1 mA at F3 and F4	40 min/day, 5 consecutive days	Stable Antidepressants Allowed
	Gehrman et al. (2024) [41]	USA	DSM-5, MINI	39.6 (10.0) 30/96	40.1 (10.9) 40/89	Squamous temporal bone on either side 15,000 Hz carrier; 500 and 15 Hz modulation; pulse width 33.3 μs 2 mA	40 min/day, 4 weeks	Concurrent Medication Allowed
HI-tACS								
	Wang et al. (2022) [42]	China	DSM-IV-TR, MINI	38.3 (12.2) 11/39	41.6 (12.8) 15/35	Forehead (Fpz, Fp1 and Fp2) and mastoid region of each side 77.5 Hz 15 mA	40 min/day, from Monday to Friday for 4 weeks (20 sessions)	Drug-naïve
	Zhou et al. (2024) [43]	China	DSM-5	29.4 (8.8) 8/25	27.5 (7.6) 6/27	Forehead (Fpz, Fp1 and Fp2) and mastoid region of each side 77.5 Hz 15 mA	40 min/day, from Monday to Friday for 4 weeks (20 sessions)	Add-on to Escitalopram

*Note.* Alexander et al. (2019) [40] included two intervention arms, with active tACS applied at frequencies of (a) 10 Hz and (b) 40 Hz. The numbers presented in the table reflect the total number of participants at the start of the experiment, however, due to discontinuation, the final number of participants included in the meta-analysis was lower. Data are presented as mean (SD) or number (n). EEG electrode positions are named according to the international 10-20 system. Abbreviations: Hz, hertz; mA, milliamperes; SD, standard deviation; LI-tACS, low-intensity transcranial alternating current stimulation; HI-tACS, high-intensity transcranial alternating current stimulation; DSM-IV, Diagnostic and Statistical Manual of Mental Disorders, Fourth Edition; DSM-IV-TR, DSM Fourth Edition Text Revision; DSM-5, DSM Fifth Edition; MINI, Mini International Neuropsychiatric Interview; EEG, electroencephalography.

### 3.3 Study Quality

All studies included in this analysis reported randomization and described the procedures used for generating random sequences. Adequate allocation concealment was confirmed in all studies. Regarding blinding, two studies [41,42] successfully implemented blinding of participants, personnel, and outcome assessors, indicating a low risk of bias. For one study [43], although outcome assessors were blinded, the risk of bias for blinding of participants and personnel was categorized as unclear because the device operators were aware of the allocation codes. In terms of incomplete outcome data, two studies [40,43] reported attrition rates exceeding 10%. Although these studies employed intention-to-treat analyses or imputation methods to handle missing data, the risk of attrition bias was classified as unclear given the attrition threshold. No instances of selective reporting or other potential biases were identified. The detailed justifications for the risk of bias assessment for each included study are provided in **Supplementary Table 3**. The risk of bias for each individual study is illustrated in **Supplementary Fig. 2**. A detailed evaluation of these factors and the proportions of trials categorized by low, unclear, and high risk of bias in each domain are presented in **Supplementary Fig. 3**.

### 3.4 Change of Depressive Scale Scores

**Fig. 1.**
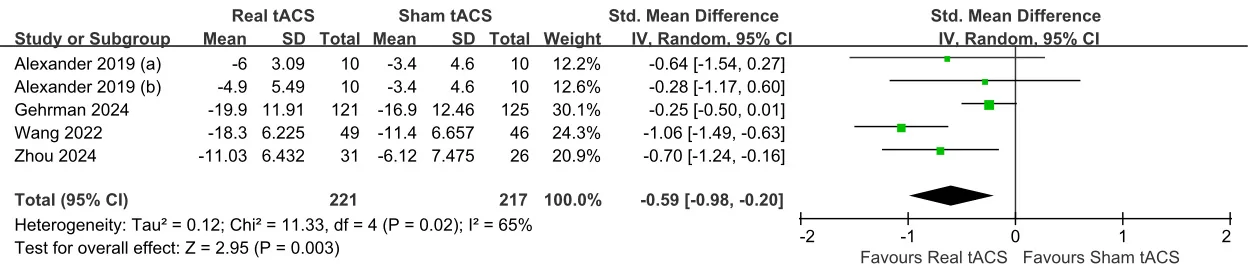
**Forest plot of the overall effect of tACS on depressive symptoms**. The plot compares the change in depressive scale scores in participants receiving active tACS versus those receiving sham stimulation across all included studies. The diamond represents the pooled standardized mean difference (SMD), indicating a significant moderate effect in favor of active tACS. Abbreviations: CI, confidence interval; IV, inverse variance; df, degrees of freedom; Std, standardized.

Three articles employed the HDRS for outcome measurement [40,42,43], while one used the Beck Depression Inventory (BDI) [41]. The primary meta-analysis of all included RCTs demonstrated that active tACS significantly reduced depressive symptoms compared to sham stimulation, yielding a moderate effect (SMD = –0.59, 95% CI: –0.98 to –0.20; Z = 2.95, *p* = 0.003) with considerable heterogeneity (I^2^ = 65%), as depicted in Fig. 1. The funnel plot for the primary outcome (**Supplementary Fig. 4**) should be interpreted with caution, given the small number of included studies [44].

**Fig. 2.**
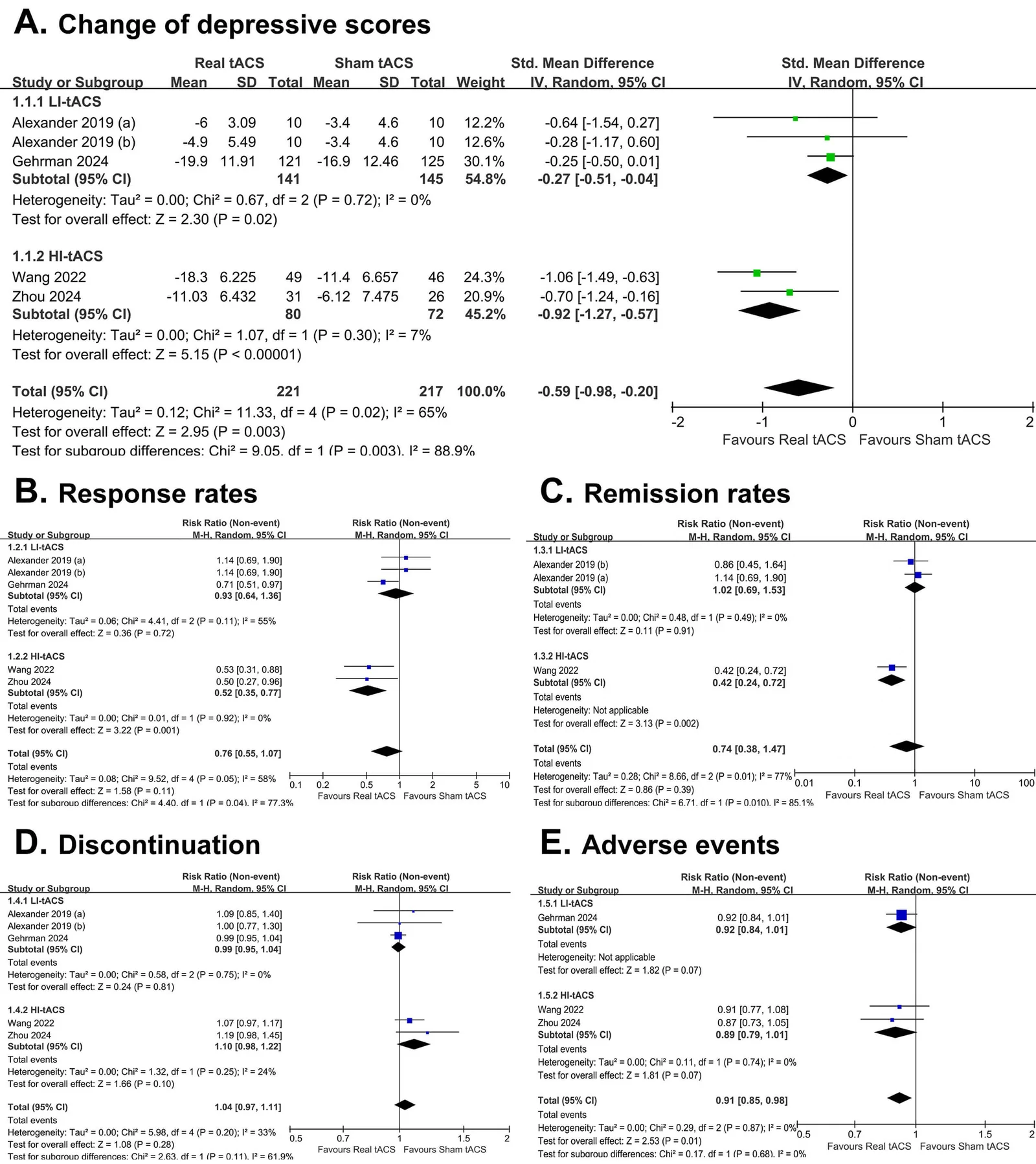
**Forest plots from the exploratory analysis of treatment outcomes based on current intensity**. These plots illustrate the preliminary findings when comparing HI-tACS and LI-tACS against sham stimulation. Outcomes include: (A) change in depressive scores, showing different effect sizes between subgroups; (B) response rates; (C) remission rates; (D) discontinuation rates; and (E) incidence of adverse events. These subgroup analyses should be interpreted with caution. Abbreviations: M-H, Mantel-Haenszel.

Following this, an exploratory subgroup analysis was conducted to investigate the potential influence of current intensity by comparing the effects of LI-tACS and HI-tACS (Fig. 2A). LI-tACS, while still demonstrating efficacy, showed a small effect (SMD = –0.27, 95% CI: –0.51 to –0.04; Z = 2.30, *p* = 0.02), whereas HI-tACS exhibited a large effect (SMD = –0.92, 95% CI: –1.27 to –0.57; Z = 5.15, *p* < 0.001). A statistically significant difference was observed between the subgroups (*p* = 0.003). The heterogeneity within the subgroups was minimal (I^2^_LI-tACS_ = 0%, I^2^_HI-tACS_ = 7%), while the heterogeneity between the subgroups was large (I^2^ = 88.9%). A funnel plot assessing publication bias is shown in **Supplementary Fig. 4**.

### 3.5 Response and Remission Rates

Within the LI-tACS group, the proportion of participants meeting the response criteria did not significantly differ from that in the control group (RR = 0.93, 95% CI: 0.64 to 1.36; Z = 0.36, *p* = 0.72, I^2^ = 55%). In contrast, a significantly higher proportion met the response criteria in the HI-tACS group compared to controls (RR = 0.52, 95% CI: 0.35 to 0.77; Z = 3.22, *p* = 0.001, I^2^ = 0%), although the overall effect of tACS on response rates was not statistically significant (RR = 0.76, 95% CI: 0.55 to 1.07; Z = 1.58, *p* = 0.11, I^2^ = 58%). A statistically significant difference was observed between subgroups (*p* = 0.04), as shown in Fig. 2B.

Similarly, remission rates did not significantly differ between the LI-tACS and control groups (RR = 1.02, 95% CI: 0.69 to 1.53; Z = 0.11, *p* = 0.91, I^2^ = 0%). However, the HI-tACS group exhibited a significantly higher remission rate than controls (RR = 0.42, 95% CI: 0.24 to 0.72; Z = 3.13, *p* = 0.002). The overall effect of tACS on remission remained nonsignificant (RR = 0.74, 95% CI: 0.38 to 1.47; Z = 0.86, *p* = 0.39, I^2^ = 77%). A statistically significant difference was observed between subgroups (*p* = 0.01), as shown in Fig. 2C.

### 3.6 Adverse Events and Discontinuation

The discontinuation rates for both active LI-tACS (RR = 0.99, 95% CI: 0.95 to 1.04; Z = 0.24, *p* = 0.81, I^2^ = 0%) and HI-tACS (RR = 1.10, 95% CI: 0.98 to 1.22; Z = 1.66, *p* = 0.10, I^2^ = 24%) did not significantly differ from those of the control group. The overall effect of tACS on discontinuation rates was not statistically significant (RR = 1.04, 95% CI: 0.97 to 1.11; Z = 1.08, *p* = 0.28, I^2^ = 33%), as shown in Fig. 2D.

No severe adverse events, such as seizures, syncope, or manic episodes, were reported in any of the studies. The most commonly reported side effects included headaches, dizziness, and tinnitus. The incidence of any reported adverse event was higher for active LI-tACS (19/126 vs. 10/129; RR = 0.92, 95% CI: 0.84 to 1.01; Z = 1.82, *p* = 0.07) and HI-tACS (16/83 vs. 8/83; RR = 0.89, 95% CI: 0.79 to 1.01; Z = 1.81, *p* = 0.07, I^2^ = 0%), although these differences were not statistically significant. However, the overall effect of tACS on the occurrence of adverse events was statistically significant (35/209 vs. 18/212; RR = 0.91, 95% CI: 0.85 to 0.98; Z = 2.53, *p* = 0.01, I^2^ = 0%), as shown in Fig. 2E.

### 3.7 Outcomes at Follow-up

Consistent with the findings at the treatment endpoint, at the 4-week follow-up, LI-tACS demonstrated a significantly greater reduction in HDRS scores (SMD = –0.97, 95% CI: –1.91 to –0.03; Z = 2.02, *p* = 0.04), although the response rates (RR = 0.76, 95% CI: 0.39 to 1.48; Z = 0.80, *p* = 0.42) and remission rates (RR = 1.58, 95% CI: 0.73 to 3.45; Z = 1.15, *p* = 0.25) were not significantly different. In contrast, HI-tACS showed a significant reduction in HDRS scores (SMD = –0.88, 95% CI: –1.21 to –0.54; Z = 5.14, *p* < 0.001), as well as significant improvements in both response rates (RR = 0.51, 95% CI: 0.36 to 0.72; Z = 3.83, *p* < 0.001) and remission rates (RR = 0.56, 95% CI: 0.40 to 0.79; Z = 3.35, *p* < 0.001). In subgroup analyses, HI-tACS showed significantly better remission rates (*p* = 0.02) compared to LI-tACS. However, no significant differences were observed between HI-tACS and LI-tACS in terms of HDRS scores (*p* = 0.85) or response rates (*p* = 0.30). These results are shown in **Supplementary Figs. 5–7**.

## 4. Discussion

To the best of our knowledge, this is the first meta-analysis examining the influence of stimulus parameters on the treatment outcomes of tACS. Our results suggest that HI-tACS may be more effective than LI-tACS in the treatment of MDD, without causing significantly more adverse effects.

This research prioritized the use of continuous data rather than dichotomous data, as dichotomizing depressive scale scores can lead to information loss, reduced statistical power, and an increased risk of Type I errors [45]. Furthermore, we emphasized changes from baseline rather than relying solely on endpoint values. As recommended by the Cochrane Handbook [31], this approach reduces between-person variability and is considered more statistically powerful.

To bridge the gap between statistical findings and clinical relevance, the Minimal Clinically Important Difference (MCID) characterizes the smallest improvement considered meaningful by patients [4]. In the context of depression trials, the National Institute for Health and Care Excellence (NICE) previously adopted a reduction of approximately 3 points on the HDRS as a criterion for clinical significance, which is cited to correspond to an SMD of 0.50 [4,46,47]. Application of these benchmarks reveals a potential divergence in clinical utility. Although LI-tACS achieved statistical significance, its observed effect size (SMD = –0.27) appears to fall below the 0.50 threshold suggested for clinical significance, indicating that its practical therapeutic value as a monotherapy might be relatively modest [4]. Conversely, HI-tACS demonstrated a large effect size (SMD = –0.92) that substantially exceeds this proposed MCID criterion [4]. These initial results tentatively suggest that employing an adequate current intensity may be a critical factor in driving clinical recovery that is perceptible to patients.

A previous meta-analysis by Zheng et al. (2023) [14] reported the efficacy of tACS in treating MDD. However, substantial heterogeneity between studies (I^2^ = 90%) limited the reliability of its conclusions. Furthermore, the inclusion of articles from the same research center may have led to repeated calculations. By refining our methodology and incorporating updated studies with larger sample sizes, we observed low heterogeneity within the subgroups (I^2^_LI-tACS_ = 0%, I^2^_HI-tACS_ = 7%), suggesting that stimulus intensity may be an important factor influencing treatment outcomes [48].

Our rationale for the >7 mA stratification is grounded in converging biophysical evidence suggesting a nonlinear threshold for transcranial stimulation. Vöröslakos et al. (2018) [17] demonstrated that scalp-applied currents must produce an intracranial electric field of at least 1 V/m to effectively entrain neuronal spiking, a threshold that conventional low-intensity (<2 mA) protocols rarely achieve in deep brain regions. Furthermore, immediate neurophysiological effects of transcranial electrical stimulation have been shown to be strictly intensity-dependent [18,28]. This supports our hypothesis that LI-tACS may have failed to reach the physiological threshold necessary for therapeutic efficacy in MDD, whereas HI-tACS provided sufficient field strength to modulate the relevant pathological circuits. Our rationale for categorizing studies by a >7 mA threshold stems from the physiological findings of Shan et al. (2023) [28], which suggest that higher current intensities might be required to effectively modulate deep brain structures crucial to MDD pathophysiology.

With the application of higher intensities, safety concerns are a common consideration. Our findings suggest that HI-tACS did not result in serious adverse events, such as seizures, and no significant differences were observed between subgroups regarding reported side effects. From an ethical and safety perspective, it is important to contextualize the intensity used in HI-tACS. The 15 mA current used in the included HI-tACS studies is substantially lower than the 800–900 mA constant current typically employed in electroconvulsive therapy (ECT) [49].

While our findings support the superiority of higher intensities, the >7 mA threshold should be interpreted within the context of the specific montages used in the included trials. Intracranial findings do not directly translate to effective electric fields due to the complex impedance of the scalp–skull–brain volume conductor. Finite-element electric-field modeling (FEM) studies suggest that to reach the required intracranial electric field of 1 V/m for neuronal entrainment, scalp currents significantly higher than conventional doses are necessary [17,19]. However, the exact field strength is highly sensitive to individual anatomical variations and electrode impedance ranges. Therefore, >7 mA should not be viewed as a rigid cutoff but rather as a range where the probability of achieving biologically effective field strengths significantly increases compared to traditional 1–2 mA protocols.

The evolution of ECT itself provides a valuable precedent. Sackeim et al. (1993) [50] established a non-negotiable boundary: stimulation must substantially exceed the individual’s seizure threshold to remain therapeutically effective. This finding was subsequently reinforced by large-scale trials demonstrating that high-dose protocols were necessary to achieve remission rates comparable to gold-standard bilateral placement [51,52]. Crucially, the field advanced by refining stimulus waveforms—such as the transition to ultrabrief pulses—to maintain this high suprathreshold intensity while minimizing cognitive side effects [53,54]. Our investigation into tACS intensity can be seen as part of this broader effort in neuromodulation to optimize stimulation parameters to maximize the risk-benefit ratio (**Supplementary Table 4**).

Moreover, the electrode montages used in these studies, with placements on the forehead and mastoid, were distant from the temporal and motor cortices, further mitigating risk. Notably, given that the overall effect of adverse events combining the HI-tACS and LI-tACS groups was significant (Fig. 2E), the lack of significant differences within the individual subgroups may be due to small sample sizes. Thus, the safety profile of HI-tACS should be considered preliminary, and future clinical trials must continue to employ rigorous monitoring of adverse effects [55,56].

We also examined the potential influence of concomitant treatments. The HI-tACS subgroup comprised one study on drug-naïve patients [42] and one add-on study with concurrent antidepressants [43]. Both protocols yielded robust effect sizes, implying that the efficacy of high-intensity stimulation might be relatively independent of medication status. In contrast, LI-tACS trials, which consistently allowed stable concomitant medication, failed to show comparable benefits.

Limitations of this study should be acknowledged. Despite expanding our search strategy to include controlled vocabularies and clinical trial registries to capture grey literature, the number of eligible high-quality RCTs remains small. This scarcity reflects the rigorous adherence to inclusion criteria and the nascent stage of high-intensity tACS research. We prioritized the avoidance of double-counting bias by rigorously excluding earlier reports from the same centers. Unlike a previous synthesis, which incorporated overlapping datasets from the same patient populations [14], we adhered to the principle that inflating the sample size with non-independent data leads to spurious precision and artificial heterogeneity [29,30]. However, the limited number of included studies imposes inherent restrictions on the stability of effect size estimates and the precise estimation of heterogeneity [57]. Consequently, the findings, particularly regarding the superiority of HI-tACS, should be interpreted as exploratory and hypothesis-generating rather than confirmatory. Although leading authorities in meta-analysis have noted that syntheses are valid with as few as two studies when the aim is to identify dispersion patterns [34,58], future large-scale trials are essential to verify these preliminary signals. 

There was substantial heterogeneity between the two subgroups. Specifically, we must acknowledge a complete overlap between current intensity and geographical region in the included studies: all HI-tACS trials were conducted in China, while all LI-tACS trials were in the USA. Although anatomical differences between Caucasian and Chinese head models result in only minor variations in cortical current flow [59], we cannot exclude the possibility that regional factors such as differences in patient baseline characteristics, healthcare systems, or placebo response rates between Asian and Western populations [60] contributed to the observed differences. This confounding factor reinforces the need to interpret our findings as preliminary.

Furthermore, all included RCTs were single-center studies, which may affect the stability of the findings across different clinical contexts. Additionally, our primary analysis of the multi-arm Alexander et al. (2019) study [40] involved two comparisons against a shared control group, creating a unit-of-analysis error. We therefore performed a sensitivity analysis (see **Supplementary Fig. 8** and **Supplementary Fig. 9**) using the splitting the shared control group method [61,62,63], which confirmed the robustness of our central finding. Despite our rigorous adherence to the Cochrane Handbook for study selection and quality assessment, these issues could not be fully addressed.

In addition to stimulus intensity, other critical parameters were not consistently controlled. For instance, studies used different frequencies, which may engage distinct neurobiological mechanisms. While 10 Hz stimulation is typically intended to entrain endogenous alpha oscillations [64], the 77.5 Hz protocol used in HI-tACS has been recently shown to induce brain-wide activation in stress and reward-related nuclei in a human modeling study [65]. This suggests that frequency differences likely contribute to the observed heterogeneity in clinical outcomes [66]. Future research should systematically investigate frequency-specific effects, perhaps guided by network-level hypotheses, such as using alpha-band tACS to modulate the Default Mode Network or gamma-band tACS to target the Salience Network [15]. Similarly, advances in stimulation technology, such as high-definition tACS (HD-tACS) and bifocal montages, offer the potential to more precisely target specific cortical nodes or modulate inter-regional connectivity, providing a path beyond the broader stimulation used in the studies included in this review [15]. Finally, while we adopted the standard consensus definition for remission (HDRS ≤7) [67], we acknowledge the ongoing debate in the field regarding whether a more stringent threshold is needed to truly capture a full symptomatic and functional recovery [68].

Therefore, this exploratory meta-analysis suggests that tACS is a promising treatment, but the conclusions regarding intensity should be considered hypothesis-generating and interpreted with caution. Using the Grading of Recommendations Assessment, Development and Evaluation (GRADE) approach, the certainty of evidence for the primary outcome was graded as Low. This rating reflects downgrades for inconsistency and imprecision. Given that LI-tACS (<7 mA) consistently showed negligible effects and low heterogeneity (I^2^_LI-tACS_ = 0%), our findings serve as a rational alert for future research resource allocation. We suggest that future trials should prioritize parameters that are physiologically grounded rather than repeating low-intensity designs. Furthermore, rigorous multi-arm RCTs are urgently needed to definitively verify or falsify the hypothesis that intensity, rather than regional variance, is the true determinant of efficacy.

## 5. Conclusions

In conclusion, this systematic and exploratory meta-analysis suggests that tACS is a promising and effective treatment for MDD. Our exploratory findings offer preliminary data consistent with the hypothesis that current intensity may be a critical parameter, with HI-tACS potentially offering greater therapeutic benefits than LI-tACS. However, it is crucial to emphasize that due to the limited number of studies and the co-variation of intensity with other variables (such as stimulation frequency and geographical region), these subgroup findings should be interpreted as hypothesis-generating rather than confirmatory. The current evidence does not yet permit definitive causal attribution regarding intensity alone. The results should be interpreted with caution, serving primarily as a rationale for future, more definitive research. Large-scale, multicenter, and well-controlled trials are essential to confirm these preliminary observations and isolate the specific contributions of intensity versus frequency for MDD.

## Data Availability

The datasets used and analysed during the current study are available from the corresponding authors on reasonable request.
