# Supplementary material for: Efficacy of Transcranial Alternating Current Stimulation for Major Depressive Disorder and the Role of Intensity: A Systematic Review and Exploratory Meta-Analysis
Source: Alpha Psychiatry. 2026 Jul 31;27(4):47383. doi: 10.31083/AP47383 (PMC13540047; doi:10.31083/AP47383)
Supplement: Supplementary file 1 [file 2757-8038-27-4-47383-s1.zip › Supplementary Material.pdf]

SUPPLEMENTARY INFORMATION

Efficacy of Transcranial Alternating Current Stimulation for Major Depressive Disorder and the Role of Intensity: A Systematic Review and Exploratory Meta-Analysis

Qi *et al.*

Contents	Page
**Supplementary Tables**	3
**Supplementary Table 1.** PRISMA 2020 Checklist.	3
**Supplementary Table 2.** Search strategy details.	6
**Supplementary Table 3.** Assessment of study quality.	7
**Supplementary Table 4.** Conceptual Comparison of Parameters and Theoretical Rationale Across Neuromodulation	8
**Supplementary Figures**	9
**Supplementary Fig. 1.** PRISMA 2020 flow diagram illustrating the search results and study selection process.	9
**Supplementary Fig. 2.** Risk of bias assessment for individual studies.	10
**Supplementary Fig. 3.** Risk of bias summary for all included studies.	11
**Supplementary Fig. 4.** Funnel plot of the main outcome.	12
**Supplementary Fig. 5.** Forest plot of the reduction in depressive symptom scores at follow-up.	13
**Supplementary Fig. 6.** Forest plot of response rates at follow-up.	14
**Supplementary Fig. 7.** Forest plot of remission rates at follow-up.	15
**Supplementary Fig. 8.** Sensitivity analysis of the overall effect of tACS on depressive symptoms.	16
**Supplementary Fig. 9.** Sensitivity analysis of the subgroup analysis by current intensity.	17
**Supplementary References**	18

Supplementary Tables

Supplementary Table 1. PRISMA 2020 Checklist.

Section and Topic	Item #	Checklist item	Location where item is reported
**TITLE**			
Title	1	Identify the report as a systematic review.	#1
**ABSTRACT**			
Abstract	2	See the PRISMA 2020 for Abstracts checklist.	#1
**INTRODUCTION**			
Rationale	3	Describe the rationale for the review in the context of existing knowledge.	#2
Objectives	4	Provide an explicit statement of the objective(s) or question(s) the review addresses.	#2
**METHODS**			
Eligibility criteria	5	Specify the inclusion and exclusion criteria for the review and how studies were grouped for the syntheses.	#2-3
Information sources	6	Specify all databases, registers, websites, organisations, reference lists and other sources searched or consulted to identify studies. Specify the date when each source was last searched or consulted.	#3, Figure S1
Search strategy	7	Present the full search strategies for all databases, registers and websites, including any filters and limits used.	#3, Table S2
Selection process	8	Specify the methods used to decide whether a study met the inclusion criteria of the review, including how many reviewers screened each record and each report retrieved, whether they worked independently, and if applicable, details of automation tools used in the process.	#3
Data collection process	9	Specify the methods used to collect data from reports, including how many reviewers collected data from each report, whether they worked independently, any processes for obtaining or confirming data from study investigators, and if applicable, details of automation tools used in the process.	#3
Data items	10a	List and define all outcomes for which data were sought. Specify whether all results that were compatible with each outcome domain in each study were sought (e.g. for all measures, time points, analyses), and if not, the methods used to decide which results to collect.	#3
	10b	List and define all other variables for which data were sought (e.g. participant and intervention characteristics, funding sources). Describe any assumptions made about any missing or unclear information.	#3
Study risk of bias assessment	11	Specify the methods used to assess risk of bias in the included studies, including details of the tool(s) used, how many reviewers assessed each study and whether they worked independently, and if applicable, details of automation tools used in the process.	#4

Section and Topic	Item #	Checklist item	Location where item is reported
Effect measures	12	Specify for each outcome the effect measure(s) (e.g. risk ratio, mean difference) used in the synthesis or presentation of results.	#3
Synthesis methods	13a	Describe the processes used to decide which studies were eligible for each synthesis (e.g. tabulating the study intervention characteristics and comparing against the planned groups for each synthesis (item #5)).	#3
	13b	Describe any methods required to prepare the data for presentation or synthesis, such as handling of missing summary statistics, or data conversions.	#3
	13c	Describe any methods used to tabulate or visually display results of individual studies and syntheses.	Fig. 1-2, S5-S9
	13d	Describe any methods used to synthesize results and provide a rationale for the choice(s). If meta-analysis was performed, describe the model(s), method(s) to identify the presence and extent of statistical heterogeneity, and software package(s) used.	#3-4
	13e	Describe any methods used to explore possible causes of heterogeneity among study results (e.g. subgroup analysis, meta-regression).	#3
	13f	Describe any sensitivity analyses conducted to assess robustness of the synthesized results.	Fig. S8-S9
Reporting bias assessment	14	Describe any methods used to assess risk of bias due to missing results in a synthesis (arising from reporting biases).	#4, Fig. S4
Certainty assessment	15	Describe any methods used to assess certainty (or confidence) in the body of evidence for an outcome.	#9
**RESULTS**			
Study selection	16a	Describe the results of the search and selection process, from the number of records identified in the search to the number of studies included in the review, ideally using a flow diagram.	#4, Fig. S1
	16b	Cite studies that might appear to meet the inclusion criteria, but which were excluded, and explain why they were excluded.	#4
Study characteristics	17	Cite each included study and present its characteristics.	#4-5
Risk of bias in studies	18	Present assessments of risk of bias for each included study.	#4, Table S3
Results of individual studies	19	For all outcomes, present, for each study: (a) summary statistics for each group (where appropriate) and (b) an effect estimate and its precision (e.g. confidence/credible interval), ideally using structured tables or plots.	#4-7, Fig. 1, Fig. S5-S7
Results of	20a	For each synthesis, briefly summarise the characteristics and risk of bias among contributing studies.	#4-5

Section and Topic	Item #	Checklist item	Location where item is reported
syntheses	20b	Present results of all statistical syntheses conducted. If meta-analysis was done, present for each the summary estimate and its precision (e.g. confidence/credible interval) and measures of statistical heterogeneity. If comparing groups, describe the direction of the effect.	#4-7, Fig. 1, Fig. S5-S7
	20c	Present results of all investigations of possible causes of heterogeneity among study results.	#4-9, Fig. 1, Fig. S5-S7
	20d	Present results of all sensitivity analyses conducted to assess the robustness of the synthesized results.	NA
Reporting biases	21	Present assessments of risk of bias due to missing results (arising from reporting biases) for each synthesis assessed.	#4, Table S3
Certainty of evidence	22	Present assessments of certainty (or confidence) in the body of evidence for each outcome assessed.	#9, Fig. 1, Fig. S5-S7
**DISCUSSION**			
Discussion	23a	Provide a general interpretation of the results in the context of other evidence.	#6-8
	23b	Discuss any limitations of the evidence included in the review.	#8-9
	23c	Discuss any limitations of the review processes used.	#8-9
	23d	Discuss implications of the results for practice, policy, and future research.	#8-9
**OTHER INFORMATION**			
Registration and protocol	24a	Provide registration information for the review, including register name and registration number, or state that the review was not registered.	#2
	24b	Indicate where the review protocol can be accessed, or state that a protocol was not prepared.	#2
	24c	Describe and explain any amendments to information provided at registration or in the protocol.	NA
Support	25	Describe sources of financial or non-financial support for the review, and the role of the funders or sponsors in the review.	#10
Competing interests	26	Declare any competing interests of review authors.	#10
Availability of data, code and other materials	27	Report which of the following are publicly available and where they can be found: template data collection forms; data extracted from included studies; data used for all analyses; analytic code; any other materials used in the review.	NA

Note. Adapted from Page et al. (2021).

Supplementary Table 2. Detailed search strategy and results for each database.

Database	Search Strategy		Results
PubMed	#1	"Depressive Disorder"[Mesh] OR "Depressive Disorder, Major"[Mesh] OR "depression":ti,ab,kw OR "MDD":ti,ab,kw	551,995
	#2	"Transcranial Electrical Stimulation"[Mesh] OR "transcranial alternating current stimulation":ti,ab,kw OR "tACS":ti,ab,kw	2,627
	#3	#1 AND #2	**116**
Embase	#1	'depression'/exp OR 'depression' OR 'depressive disorder'/exp OR 'depressive disorder' OR 'major clinical depression' OR 'major depression':ab,ti OR 'depressive disorder':ab,ti OR 'mdd':ab,ti	1,118,856
	#2	'transcranial alternating current stimulation'/exp OR 'transcranial alternating current stimulation':ab,ti OR 'tacs':ab,ti OR (('alternating' NEAR/3 'current stimulation'):ab,ti)	4,984
	#3	#1 AND #2	**355**
Web of Science	#1	TS=("Major Depressive Disorder" OR "Depression" OR "Depressive Disorder" OR "MDD" OR "Unipolar Depression" OR "Affective Disorder")	678,603
	#2	TS=("Transcranial Alternating Current Stimulation" OR "tACS" OR "Alternating Current Stimulation" OR "cranial alternating current stimulation")	3,165
	#3	#1 AND #2	**192**
The Cochrane Library	#1	[mh "Depressive Disorder, Major"] OR "major depressive disorder":ti,ab,kw OR "major depression":ti,ab,kw	18,326
	#2	[mh "Transcranial Electrical Stimulation"] OR "transcranial alternating current stimulation":ti,ab,kw OR "tACS":ti,ab,kw	971
	#3	#1 AND #2	**51**
ClinicalTrials.gov	#1	AREA[ConditionSearch](Major Depressive Disorder OR Depression)	11,970
	#2	AREA[InterventionSearch](Transcranial Alternating Current Stimulation OR tACS)	306
	#3	#1 AND #2	**30**
WHO ICTRP	"depression AND tACS" OR "depressive AND transcranial alternating current stimulation"		**6**

Note. Latest updated search was conducted on January 5, 2026. Three major bibliographic databases (Embase, PubMed, and Web of Science) and three registration databases (The Cochrane Library, ClinicalTrials.gov, and WHO ICTRP) were searched. The search strategies were adapted to the specific syntax of each database to ensure maximum sensitivity and specificity.

Supplementary Table 3. Assessment of study quality.

Study	Random sequence generation	Allocation concealment	Blinding of participants and personnel	Blinding of outcome assessment	Incomplete outcome data	Selective reporting	Other bias
Alexander et al. (2019)	Randomized study codes prepared by a member of the research lab, who was not involved in the study otherwise.	Stimulation protocol controlled by a custom Matlab interface based on the study code.	All authors and study team members were blinded to group assignments until the study's completion.	All authors and study team members were blinded to group assignments until the study's completion.	Attrition rate 18.8% (>10%). ITT analysis employed to handle missing data.	All predefined outcome measures were reported.	No other sources of bias identified.
Gehrman et al. (2024)	No restrictions on randomization for the initial 250 participants; excess randomizations beyond 250 were balanced in blocks of 10.	Randomization performed by an unblinded investigator who had no direct contact with participants.	Not explicitly mentioned.	Not explicitly mentioned.	Missing data was minimal and did not impact the results.	All predefined outcome measures were reported.	No other sources of bias identified.
Wang et al. (2022)	According to a computer-generated list of random numbers.	Randomization based on a computer-generated list of random numbers.	Participants were assigned a group via sealed envelopes containing randomization codes.	Blinding was maintained for participants, staff, and assessors to the group allocation.	Low attrition rate . Data were analyzed using the ITT principle with worst-case imputation.	All predefined outcome measures were reported	No other sources of bias identified.
Zhou et al. (2024)	Randomization sequence generated using a random number table in SAS 9.4 by a statistician not involved in the trial.	Group assignments were placed in sealed envelopes and opened by researchers at enrollment.	Device operators aware of allocation codes; participants and outcome assessors remained blinded.	Blinding maintained for both participants and operators regarding the type of stimulation.	Attrition rate 13.6% (>10%). ITT analysis employed to handle missing data.	All predefined outcome measures were reported	No other sources of bias identified.

Note. ITT: Intention-to-Treat analysis; SAS: Statistical Analysis System.

Supplementary Table 4. Conceptual Comparison of Parameters and Theoretical Rationale Across Neuromodulation Technologies (Peterchev et al., 2010; Deng et al., 2011; Sackeim, 2017; Pan et al., 2023).

Modality	Typical Current	Primary Therapeutic Rationale
Historical ECT (Sine-wave)	Variable (Constant Voltage)	Induction of a generalized seizure
Modern ECT (Brief-pulse)	800 to 900 mA (Constant Current)	Induction of a generalized seizure with minimal energy to reduce side effects
LI-tACS	~2 mA	Modulation of cortical neural oscillations
HI-tACS	~15 mA	Modulation of cortical and deep structure neural oscillations

Note. ECT: Electroconvulsive Therapy; LI-tACS: Low-Intensity Transcranial Alternating Current Stimulation; HI-tACS: High-Intensity Transcranial Alternating Current Stimulation; mA: milliamperes.

Supplementary Figures

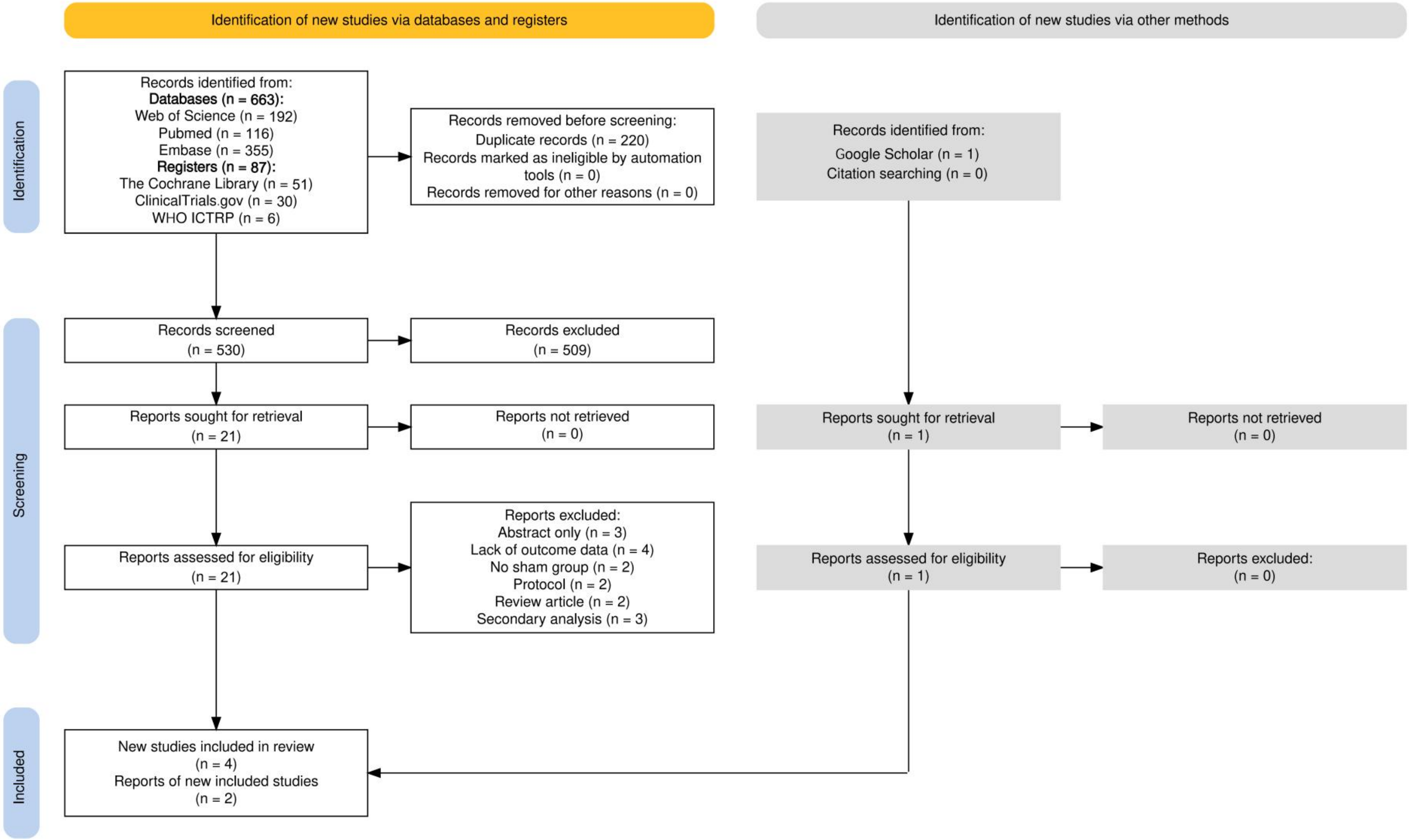

Supplementary Fig. 1. PRISMA 2020 flow diagram illustrating the search results and study selection process. The flowchart complied with PRISMA 2020 and was generated using the method provided by Haddaway et al. (2022).

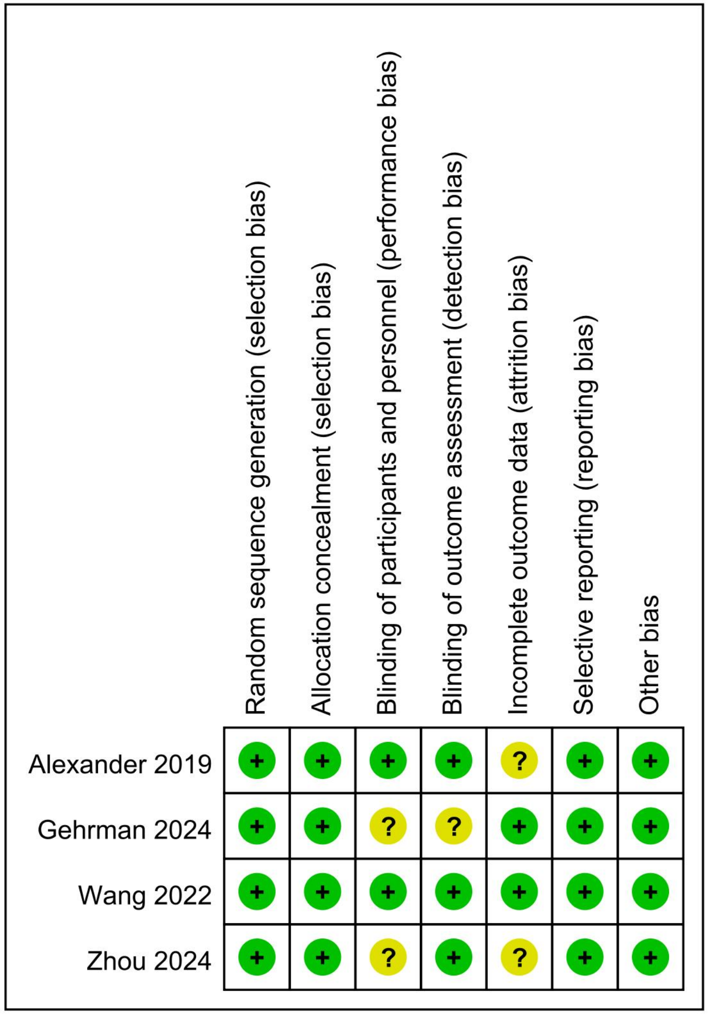

Supplementary Fig. 2. Risk of bias assessment for individual studies.

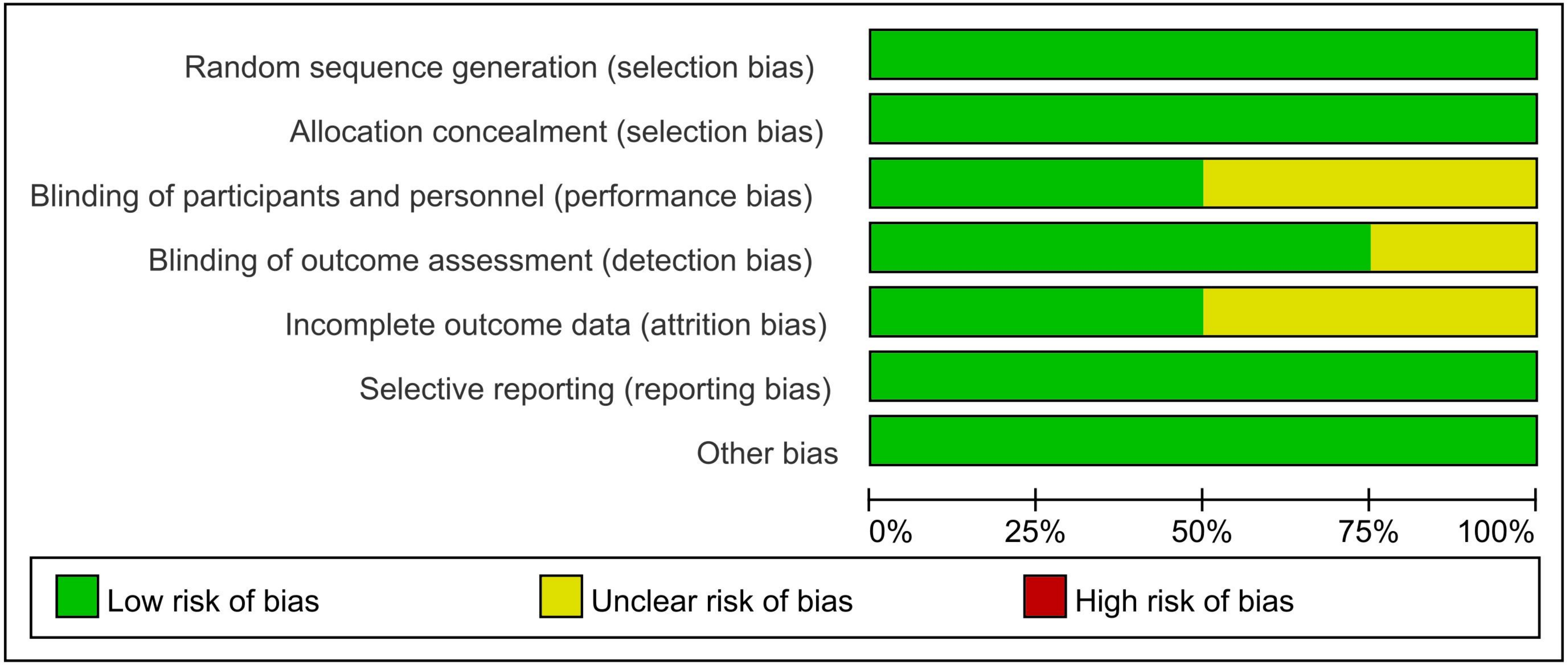

Supplementary Fig. 3. Risk of bias summary for all included studies.

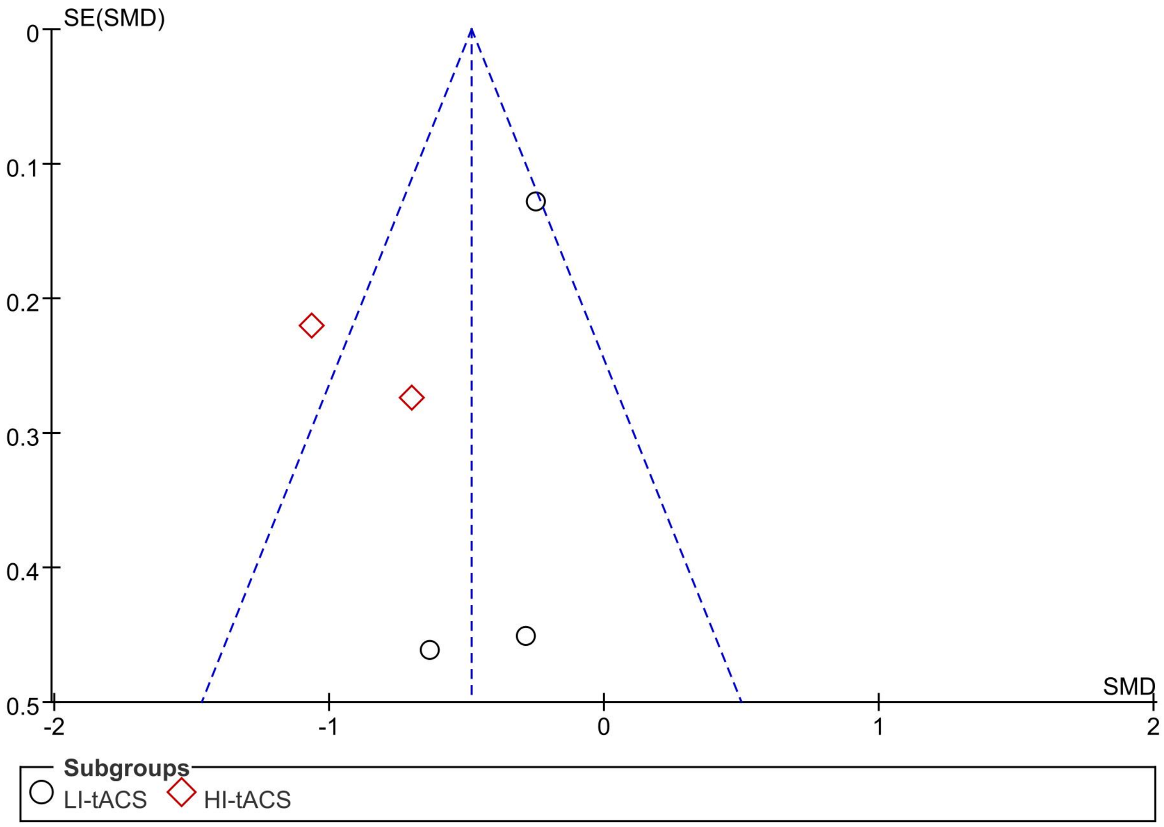

Supplementary Fig. 4. Funnel plot of the main outcome. Abbreviations: SE: Standard Error; SMD: Standardized Mean Difference.

Study or Subgroup	Real tACS Mean	Real tACS SD	Real tACS Total	Sham tACS Mean	Sham tACS SD	Sham tACS Total	Weight	Std. Mean Difference IV, Random, 95% CI
**2.1.1 LI-tACS**								
Alexander 2019 (a)	-8.67	5.32	9	-5.11	7.61	9	10.2%	-0.52 [-1.46, 0.43]
Alexander 2019 (b)	-5.2	5.67	10	5.11	7.61	9	8.4%	-1.48 [-2.52, -0.44]
**Subtotal (95% CI)**			**19**			**18**	**18.6%**	**-0.97 [-1.91, -0.03]**
Heterogeneity: Tau² = 0.21; Chi² = 1.80, df = 1 (P = 0.18); I² = 44%								
Test for overall effect: Z = 2.02 (P = 0.04)								
**2.1.2 HI-tACS**								
Wang 2022	-17.3	6.891	49	-10.9	5.682	46	49.8%	-1.00 [-1.43, -0.57]
Zhou 2024	-11.33	7.256	31	-6.42	6.931	26	31.6%	-0.68 [-1.22, -0.14]
**Subtotal (95% CI)**			**80**			**72**	**81.4%**	**-0.88 [-1.21, -0.54]**
Heterogeneity: Tau² = 0.00; Chi² = 0.84, df = 1 (P = 0.36); I² = 0%								
Test for overall effect: Z = 5.14 (P < 0.00001)								
**Total (95% CI)**			**99**			**90**	**100.0%**	**-0.89 [-1.19, -0.59]**
Heterogeneity: Tau² = 0.00; Chi² = 2.67, df = 3 (P = 0.44); I² = 0%								
Test for overall effect: Z = 5.78 (P < 0.00001)								
Test for subgroup differences: Chi² = 0.03, df = 1 (P = 0.85), I² = 0%								

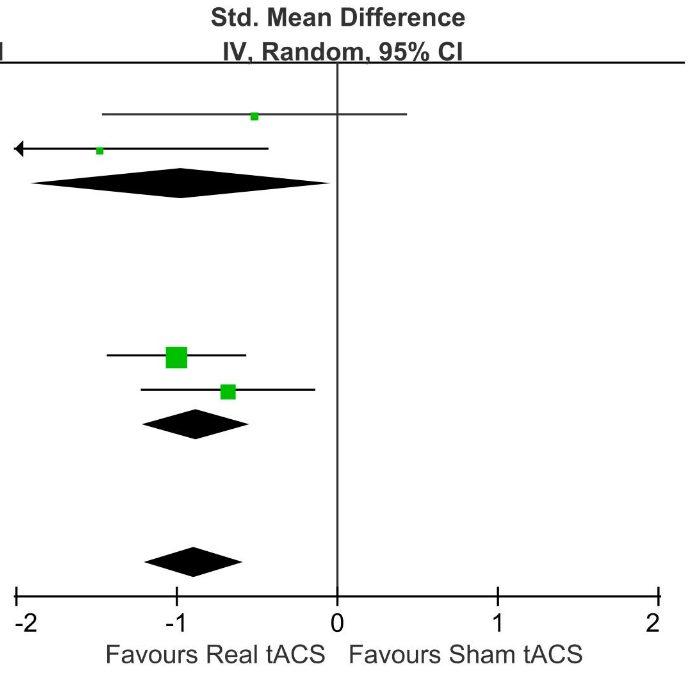

Supplementary Fig. 5. Forest plot of the reduction in depressive symptom scores at follow-up. Abbreviations: CI: Confidence Interval; IV: Inverse Variance; SD: Standard Deviation; df: degrees of freedom; SMD: Standardized Mean Difference.

Study or Subgroup	Real tACS Events	Real tACS Total	Sham tACS Events	Sham tACS Total	Weight	Risk Ratio (Non-event) M-H, Random, 95% CI
**2.2.1 LI-tACS**						
Alexander 2019 (a)	5	9	4	9	10.8%	0.80 [0.31, 2.04]
Alexander 2019 (b)	6	10	4	9	10.3%	0.72 [0.28, 1.88]
**Subtotal (95% CI)**		**19**		**18**	**21.0%**	**0.76 [0.39, 1.48]**
Total events	11		8			
Heterogeneity: Tau² = 0.00; Chi² = 0.02, df = 1 (P = 0.88); I² = 0%						
Test for overall effect: Z = 0.80 (P = 0.42)						
**2.2.2 HI-tACS**						
Wang 2022	31	49	13	46	56.1%	0.51 [0.34, 0.77]
Zhou 2024	22	31	11	26	22.9%	0.50 [0.27, 0.96]
**Subtotal (95% CI)**		**80**		**72**	**79.0%**	**0.51 [0.36, 0.72]**
Total events	53		24			
Heterogeneity: Tau² = 0.00; Chi² = 0.00, df = 1 (P = 0.96); I² = 0%						
Test for overall effect: Z = 3.83 (P = 0.0001)						
**Total (95% CI)**		**99**		**90**	**100.0%**	**0.55 [0.41, 0.75]**
Total events	64		32			
Heterogeneity: Tau² = 0.00; Chi² = 1.11, df = 3 (P = 0.77); I² = 0%						
Test for overall effect: Z = 3.77 (P = 0.0002)						
Test for subgroup differences: Chi² = 1.08, df = 1 (P = 0.30), I² = 7.6%						

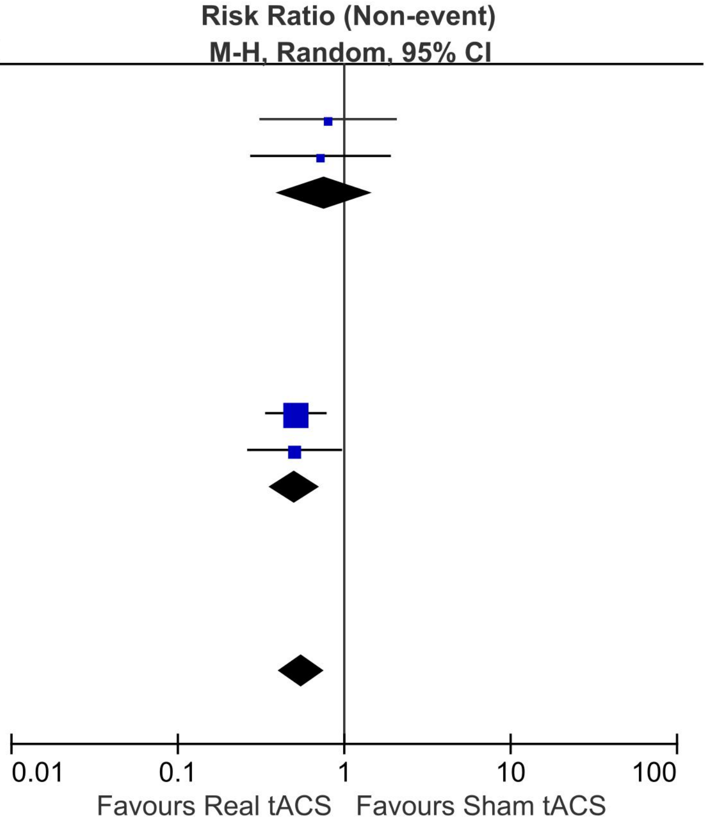

Supplementary Fig. 6. Forest plot of response rates at follow-up. Abbreviations: CI: Confidence Interval; df: degrees of freedom; RR: Risk Ratio; M-H: Mantel-Haenszel.

Study or Subgroup	Real tACS Events	Real tACS Total	Sham tACS Events	Sham tACS Total	Weight	Risk Ratio (Non-event) M-H, Random, 95% CI
**2.3.1 LI-tACS**						
Alexander 2019 (a)	4	9	6	9	26.9%	1.67 [0.56, 4.97]
Alexander 2019 (b)	5	10	6	9	26.5%	1.50 [0.49, 4.56]
**Subtotal (95% CI)**		**19**		**18**	**53.4%**	**1.58 [0.73, 3.45]**
Total events	9		12			
Heterogeneity: Tau² = 0.00; Chi² = 0.02, df = 1 (P = 0.89); I² = 0%						
Test for overall effect: Z = 1.15 (P = 0.25)						
**2.3.2 HI-tACS**						
Wang 2022	27	49	9	46	46.6%	0.56 [0.40, 0.79]
**Subtotal (95% CI)**		**49**		**46**	**46.6%**	**0.56 [0.40, 0.79]**
Total events	27		9			
Heterogeneity: Not applicable						
Test for overall effect: Z = 3.35 (P = 0.0008)						
**Total (95% CI)**		**68**		**64**	**100.0%**	**0.97 [0.43, 2.23]**
Total events	36		21			
Heterogeneity: Tau² = 0.35; Chi² = 5.91, df = 2 (P = 0.05); I² = 66%						
Test for overall effect: Z = 0.06 (P = 0.95)						
Test for subgroup differences: Chi² = 5.76, df = 1 (P = 0.02), I² = 82.6%						

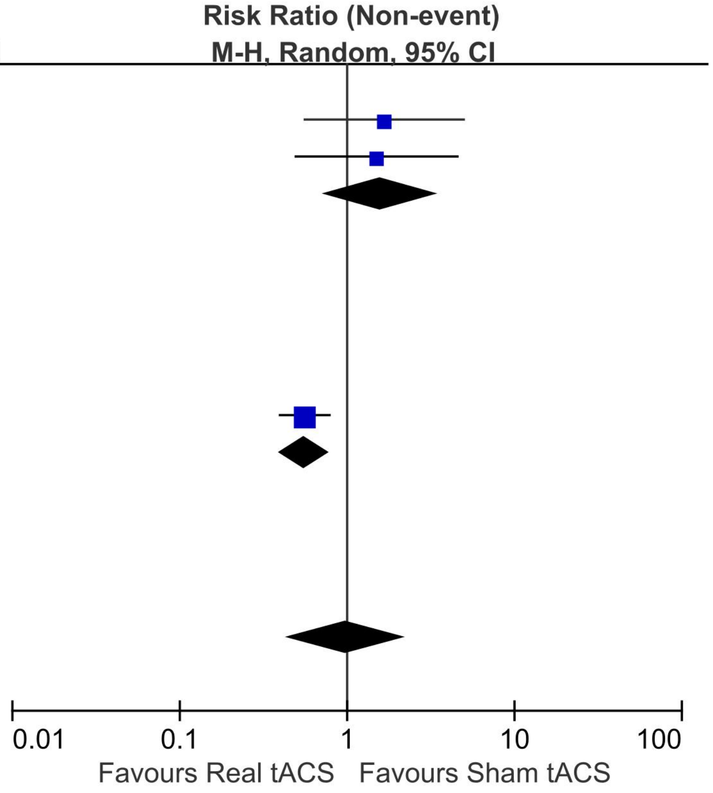

Supplementary Fig. 7. Forest plot of remission rates at follow-up. Abbreviations: CI: Confidence Interval; df: degrees of freedom; RR: Risk Ratio; M-H: Mantel-Haenszel.

	Real tACS			Sham tACS				Std. Mean Difference	Std. Mean Difference
Study or Subgroup	Mean	SD	Total	Mean	SD	Total	Weight	IV, Random, 95% CI	IV, Random, 95% CI
Alexander 2019 (a)	-6	3.09	10	-3.4	4.6	5	10.0%	-0.68 [-1.79, 0.43]	
Alexander 2019 (b)	-4.9	5.49	10	-3.4	4.6	5	10.4%	-0.27 [-1.35, 0.81]	
Gehrman 2024	-19.9	11.91	121	-16.9	12.46	125	31.6%	-0.25 [-0.50, 0.01]	
Wang 2022	-18.3	6.225	49	-11.4	6.657	46	25.7%	-1.06 [-1.49, -0.63]	
Zhou 2024	-11.03	6.432	31	-6.12	7.475	26	22.3%	-0.70 [-1.24, -0.16]	
**Total (95% CI)**			**221**			**207**	**100.0%**	**-0.60 [-1.02, -0.19]**	

Heterogeneity: Tau² = 0.12; Chi² = 11.29, df = 4 (P = 0.02); I² = 65%
Test for overall effect: Z = 2.85 (P = 0.004)

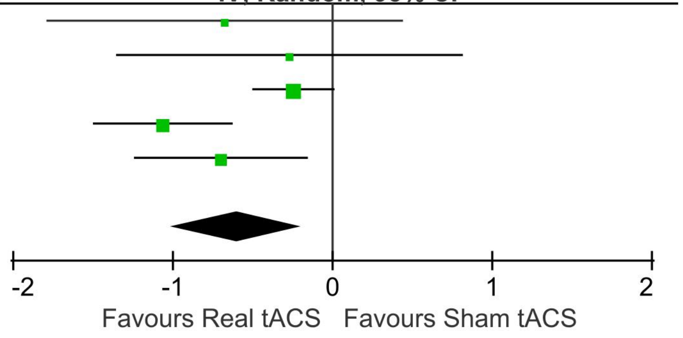

Supplementary Fig. 8. Sensitivity analysis of the overall effect of tACS on depressive symptoms. This forest plot shows a sensitivity analysis correcting the unit-of-analysis error from the multi-arm Alexander et al. (2019) trial. The analysis was conducted using the Cochrane-recommended splitting the shared control group method. The overall pooled effect remains significant, confirming the robustness of the primary finding. Abbreviations: CI: Confidence Interval; IV: Inverse Variance; SD: Standard Deviation; df: degrees of freedom; SMD: Standardized Mean Difference.

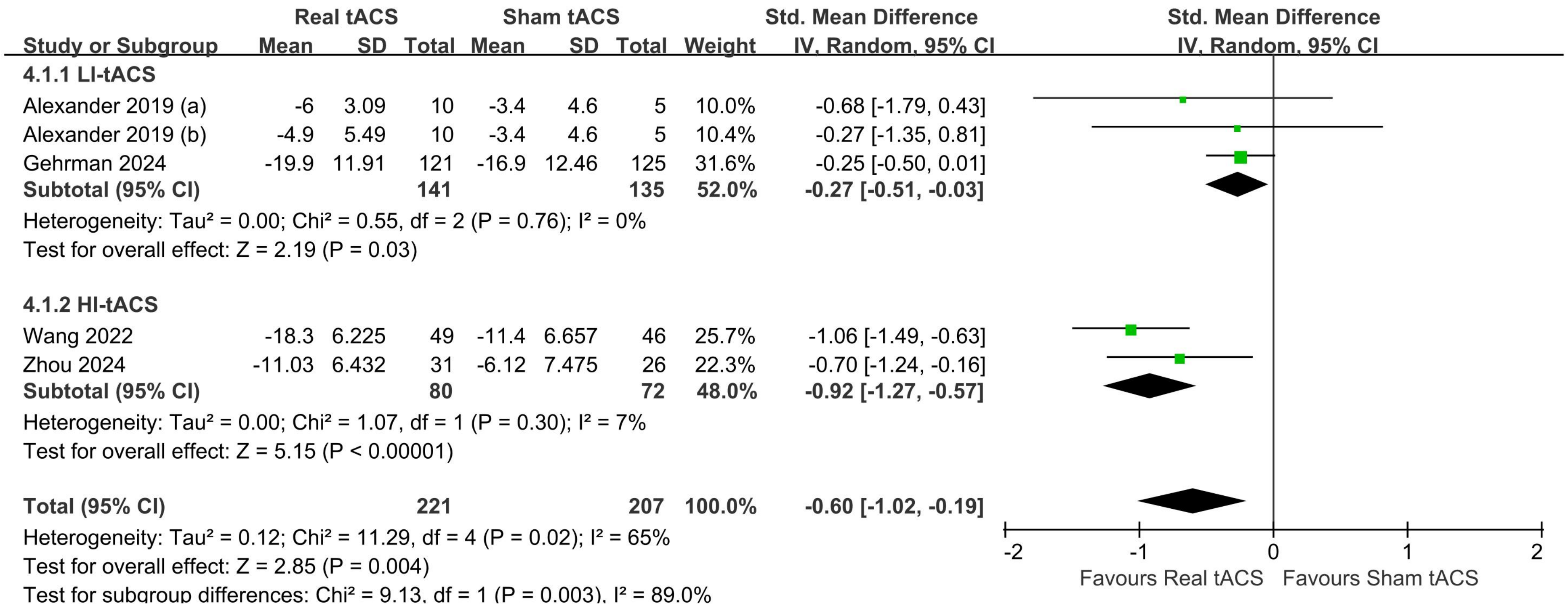

Supplementary Fig. 9. Sensitivity analysis of the subgroup analysis by current intensity. This sensitivity analysis applies the splitting the shared control group method to the Alexander et al. (2019) study within the LI-tACS subgroup. The analysis confirms the robustness of the paper's central conclusion: the effect of HI-tACS (SMD = -0.92) remains significantly larger than that of LI-tACS (SMD = -0.27), as demonstrated by the test for subgroup differences (p = 0.003). Abbreviations: CI: Confidence Interval; IV: Inverse Variance; SD: Standard Deviation; df: degrees of freedom; SMD: Standardized Mean Difference; RR: Risk Ratio; M-H: Mantel-Haenszel.

Supplementary References

Page, M.J., McKenzie, J.E., Bossuyt, P.M., Boutron, I., Hoffmann, T.C., Mulrow, C.D., Shamseer, L., Tetzlaff, J.M., Akl, E.A., Brennan, S.E., Chou, R., Glanville, J., Grimshaw, J.M., Hróbjartsson, A., Lalu, M.M., Li, T., Loder, E.W., Mayo-Wilson, E., McDonald, S., McGuinness, L.A., Stewart, L.A., Thomas, J., Tricco, A.C., Welch, V.A., Whiting, P., Moher, D., 2021. The PRISMA 2020 statement: an updated guideline for reporting systematic reviews. BMJ 372, n71. https://doi.org/10.1136/bmj.n71

Peterchev, A. V., Rosa, M. A., Deng, Z. D., Prudic, J., & Lisanby, S. H. (2010). Electroconvulsive therapy stimulus parameters: rethinking dosage. The journal of ECT, 26(3), 159–174. https://doi.org/10.1097/YCT.0b013e3181e48165

Deng, Z. D., Lisanby, S. H., & Peterchev, A. V. (2011). Electric field strength and focality in electroconvulsive therapy and magnetic seizure therapy: a finite element simulation study. Journal of neural engineering, 8(1), 016007. https://doi.org/10.1088/1741-2560/8/1/016007

Sackeim HA. Modern Electroconvulsive Therapy: Vastly Improved yet Greatly Underused. JAMA Psychiatry. 2017;74(8):779-780. doi:10.1001/jamapsychiatry.2017.1670

Pan R, Ye S, Zhong Y, Chen Q, Cai Y. Transcranial alternating current stimulation for the treatment of major depressive disorder: from basic mechanisms toward clinical applications. Front Hum Neurosci. 2023;17:1197393. Published 2023 Sep 4. doi:10.3389/fnhum.2023.1197393

Haddaway, N.R., Page, M.J., Pritchard, C.C., McGuinness, L.A., 2022. PRISMA2020: An R package and Shiny app for producing PRISMA 2020-compliant flow diagrams, with interactivity for optimised digital transparency and Open Synthesis. Campbell Syst Rev 18, e1230. https://doi.org/10.1002/cl2.1230
